# Supplementary figures and images for: Stanniocalcin-1 (STC-1), a downstream effector molecule in latanoprost signaling, acts independent of the FP receptor for intraocular pressure reduction
Source: PLoS One. 2020 May 4;15(5):e0232591. doi: 10.1371/journal.pone.0232591 (PMC7197809; doi:10.1371/journal.pone.0232591)

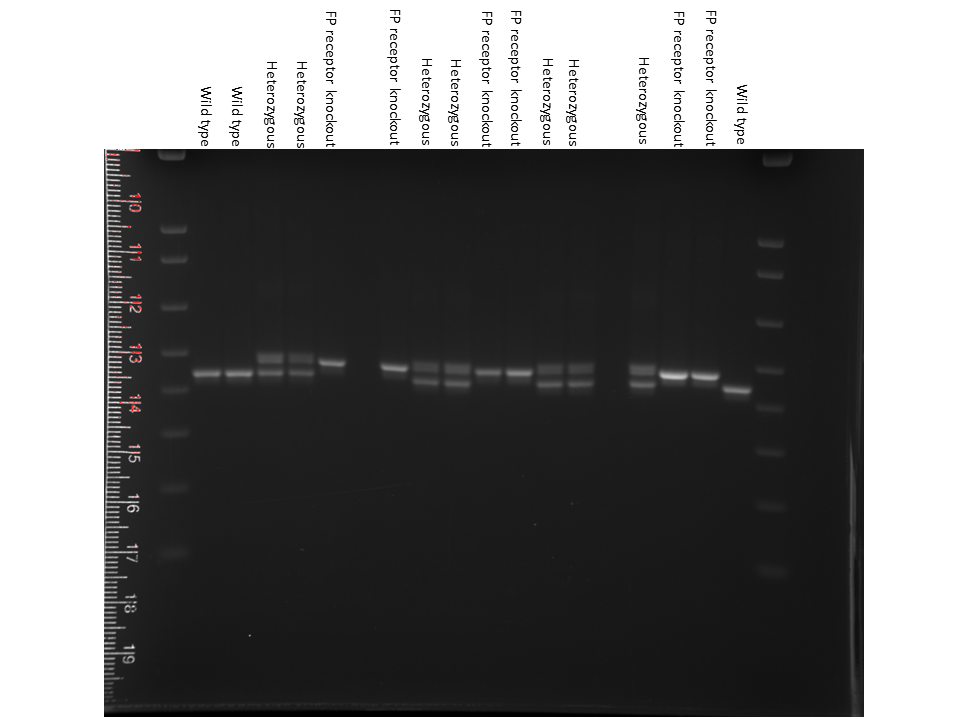

Supplement: S1 Fig — (TIF) [file pone.0232591.s001.TIF]
